# Overexpression of Contactin 1 promotes growth, migration and invasion in Hs578T breast cancer cells

**DOI:** 10.1186/s12860-018-0154-3

**Published:** 2018-04-19

**Authors:** Nan Chen, Sai He, Jie Geng, Zhang-Jun Song, Pi-Hua Han, Juan Qin, Zheng Zhao, Yong-Chun Song, Hu-Xia Wang, Cheng-Xue Dang

**Affiliations:** 1 Department of Surgical Oncology, The First Affiliated Hospital of Xi’an Jiaotong University, Xi’an, Shaanxi China; 2 Department of Breast Cancer, Shaanxi Provincial Tumor Hospital, Xi’an, Shaanxi China; 3 Department of Peripheral Vascular Disease, The First Affiliated Hospital of Xi’an Jiaotong University, Xi’an, Shaanxi China; 4 Department of Medical Oncology, Shaanxi Provincial Tumor Hospital, Xi’an, Shaanxi China

**Keywords:** CNTN1, Hs578T, Proliferation, Migration, Invasion, Breast cancer

## Abstract

**Background:**

Contactin1 (CNTN1) has been shown to play an important role in the invasion and metastasis of several tumors; however, the role of CNTN1 in breast cancer has not been fully studied. The purpose of this study is to investigate the role of CNTN1 in regulating tumor growth, migration and invasion in breast cancer.

**Results:**

To investigate its function, CNTN1 was expressed in Hs578T cells. CNTN1 expression was confirmed by western blot, immunohistochemistry and real-time RT-PCR. The effect of CNTN1 overexpression on proliferation, migration and invasion of Hs578T breast cancer cells was assessed in vitro and in vivo*.* Our results showed that CNTN1 overexpression promoted Hs578T cell proliferation, cell cycle progression, colony formation, invasion and migration. Notably, overexpression of CNTN1 in Hs578T cells enhanced the growth of mouse xenograft tumors.

**Conclusions:**

CNTN1 promotes growth, metastasis and invasion of Hs578T breast cancer cell line. Thus, therapies targeting CNTN1 may prove efficacious for breast cancer. However, further investigation is required to understand the mechanism by which CNTN1 influences proliferation, metastasis and invasion in breast cancer.

**Electronic supplementary material:**

The online version of this article (10.1186/s12860-018-0154-3) contains supplementary material, which is available to authorized users.

## Background

Breast cancer represents one of the most common cancers and is the leading cause of death in women worldwide, with an estimated 246,660 new cases of breast cancer yearly and an annual mortality over 40,000 [1–3]. Breast cancer mortalities are usually associated with spread and metastasis. Despite the remarkable improvement in therapeutic strategies targeting breast cancer, metastatic breast cancer remains incurable, prompting the need to identify drivers of metastasis in breast cancer and to develop more effective therapeutic strategies targeting these drivers.

Contactin-1 (CNTN1), a member of the immunoglobulin (Ig) family, is a glycosylphosphatidylinositol (GPI)-anchored neuronal membrane protein that functions as a neuronal cell adhesion molecule [4]. CNTN1 is abundant in the human brain and neural tissues, where it plays an important role in nervous system development [5–7]. Recently, several reports revealed that CNTN1 is an important mediator of the progression of several cancers including lung adenocarcinoma, squamous carcinoma, hepatocellular carcinoma, and gastric cancer [8–12]. CNTN1 was found to mediate tumor invasion and metastasis in lung cancer through activation of RhoA, which regulates the actin cytoskeleton and cell motility [8]. Knockdown of CNTN1 suppressed invasion and metastasis of lung adenocarcinoma [8]. The finding that CNTN1 mediates metastasis and invasion in lung cancer prompted investigation into the capacity of CNTN1 to drive invasion and metastasis of other tumors. CNTN1 expression was recently reported to be associated with lymphatic invasion and prognosis of gastric cancer [10]. Moreover, in esophageal cancer cells, vascular endothelial growth factor C (VEGF-C) was shown to enhance tumor migration and progression, which was reversed by downregulation of CNTN1 [13]. This indicates a crucial role of CNTN1 as a downstream mediator of VEGF-C-induced migration in esophageal cancer. In hepatocellular carcinoma (HCC) tissues, CNTN1 overexpression was also reported to be closely associated with aggressive clinicopathological features, suggesting that CNTN1 may be involved in tumor metastasis and invasion in HCC [12].

Accumulating evidence suggests that CNTN1 is a key player mediating invasion and metastasis of several tumors. However, the role of CNTN1 in breast cancer remains unclear. In this study, we evaluated the expression of CNTN1 in a panel of breast cancer cell lines and investigated the capacity of CNTN1 to regulate cell proliferation, migration and invasion in Hs578T breast cancer cells.

## Methods

### Cell lines and cell culture

Breast cancer cell lines MCF7-ADR, MDA-MB-468, MCF7 and Hs578T were purchased from CHI Scientific (Wuxi, China). Hs578T cells were cultured in Dulbecco’s modified Eagle’s medium (DMEM) (Gibco, USA) supplemented with 10% fetal bovine serum (FBS) (Gibco, USA) at 37C° in the presence of 5% CO_2_. The morphological changes of the cells were observed using microscope (Bio-RAD, Hercules, CA, USA).

### Vectors construction and transfection

Total RNA extraction was performed using Trizol (Invitrogen, USA). CNTN1 cDNA was then prepared by reverse transcriptase polymerase chain reaction (RT-PCR) using the isolated RNA with the following CNTN1 primer pairs: 5’-TGTTCAGCAAATTCATCCCA-3′ (forward) and 5’-TCTACCCAC TCAGGGAATGC-3′ (reverse). For CNTN1 expression vectors, Human CNTN1 DNAs (cDNA) was inserted into pEGFP-N1 vector (Clontech Laboratories, Mountain View, USA) to create CNTN1 plasmids. In addition, an empty pEGFP-N1 vector was used as a negative control.

Hs578T cells were seeded in a six-well tissue culture plate and cultured in antibiotic-free DMEM with 10% FBS for 24 h prior to transfection. Transfection of CNTN1 plasmid, and control vector were performed using Lipofectamine 2000 (Invitrogen, USA). Following incubation for 8 h at 37 °C in a CO_2_ incubator, fresh medium was added to the cells, and exposed to puromycin (800 μg/ml; Gibco Life Technologies) for two weeks. The empty vector cells and CNTN1-overexpressing cells were then routinely cloned for further analysis.

### Western blot

Protein extraction was performed using RIPA lysis buffer (Sigma, USA) with 0.2 mmol/L PMSF (Sigma) protease inhibitor. Protein quantitation was performed using BCA assay kit (Beyotime, China). Proteins were loaded in equal amounts on 10% sodium dodecyl sulfate polyacrylamide gel electrophoresis (SDS-PAGE) (Sigma). Proteins were then transferred onto a polyvinylidene fluoride membrane (Roche, Switzerland) followed by 1 h blocking with 5% nonfat dry milk (Sigma) in Tris buffer saline-Tween-20 (TBST) (Sigma). After blocking, membranes were incubated with primary rabbit antibodies against CNTN1 and B-actin (Epitomics, USA) at 4 °C overnight. The following day, the membrane was washed with TBST, then incubated for 1 h at room temperature with secondary antibody (Sigma) and washed again with TBST before detection using a chemiluminescent detection system (Beyotime, Shanghai, China). Each experiment was repeated independently three times.

### Immunostaining

Hs578T cells were fixed in 4% paraformaldehyde (Guoyao, China) after plating onto glass slides for 15 min at room temperature. Cells were then treated with 0.1% Triton-X100 (Sigma), 4% bovine serum albumin (Sigma), and incubated with rabbit anti-CNTN1 antibody (Sigma). Cells were then incubated with PE (Phycoerythrin) anti-rabbit immunoglobulin G (Sigma). Immunocytochemistry images were evaluated by three independent investigators.

### Real-time PCR

Total RNA was extracted using Trizol reagent (Invitrogen, USA) according to the manufacturer’s protocol. RNA concentration was measured using a spectrophotometer (Eppendorf, German). After RNA was reverse transcribed using MMLV reverse transcriptase and oligo (dT) primer (Sangon, China), the cDNA generated was then amplified by polymerase chain reaction (PCR) using the following primer pairs. CNTN1 S: 5’-GCCCATGACAAAGAAGAAGC-3′; CNTN1 A: 5’-CGACATGATCCCAGGTGATT-3′; B-actin S: 5’-GAAGGTGAAGGTCGGAGTC-3′; B-actin A: 5’-GAAGATGGTGATGGGATTTC-3′. Each assay was carried out in triplicate. Data were extracted and analyzed using the Realplex analysis system (Eppendorf, Germany).

### Flow cytometry

Cells (5 × 10^5^) were harvested and treated with 0.25% trypsin (Sigma) and 0.02% ethylene diamine tetraacetic acid (EDTA) (Sigma). After washing with phosphate buffered saline (PBS) twice at room temperature, the cells were resuspended in 4% paraformaldehyde (Guoyao, China) for 10 min. Cells were then treated with rectisol (Guoyao, China) at 4 °C for 15 min after washing with PBS. Then the cells were incubated with corresponding fluorescent antibodies (eBioscience, USA) for 1 h at 37 °C, and washed with PBS. Hs578T cells incubated without these agents were used as a negative control. The cells were then resuspended in 0.5 mL PBS for flow cytometry analysis. Cells were analyzed for DNA content by flow cytometry, and the cell cycle phases were analyzed using ModFit LT software (Verity Software House Inc., USA). All experiments were performed in triplicate.

### Colony formation in soft agar

Cells were suspended at a concentration of 1 × 10^3^ cells/mL in DMEM media containing FBS (10%) and agar (0.7%) (Invitrogen, USA). Then 1 mL of cell suspension was plated onto the same medium containing 1.2% agar. Colonies containing 50 or more cells were counted at day 14.

### Migration and invasion assay

Hs578T cells were digested with trypsin-EDTA (Sigma), and 5 × 10^4^ cells were suspended in serum-free medium supplemented with BSA (Sigma). Cell suspensions were seeded into the inserts of transwells (Corning, USA) and incubated at 37 °C for 48 h. After incubation, inserts were washed with PBS. Migratory cells on the underside of the membrane were fixed with 95% alcohol and stained with crystal violet (Beyotime, China). For the invasion assay, the upper chamber was pre-coated with 50 mg/L Matrigel (Sigma) prior to the addition of 1 × 10^5^ cells in serum-free medium supplemented with BSA. The number of migratory or invading cells per membrane was counted using an inverted microscope. Three fields of fixed cells were randomly selected and counted. Each experiment was repeated independently three times.

### MTT assay

Cells was seeded onto 96-well cluster cell culture plates (5 × 10^4^/mL, 200 μL per well) (Corning, USA) and incubated at 37 °C overnight before the medium was changed. After 48 h, 25 μL 3-(4,5-dimethylthiazol-2-yl)-2,5-diphenyl tetrazolium bromide (MTT) (Sigma) was added to each well, then, after 4 h, 200 μL dimethyl sulfoxide (DMSO) (Sigma) was added to each well. The optical density (OD) at 490 nm was measured using a microplate reader (Thermo, Massachusetts, USA).

### Animal study

Five week old nude male mice (athymic BALB/c mice) weighing 15 to 18 g were acquired from the Institute of Laboratory Animal science, Chinese Academy of Medical Science, Beijing, China. The mice were kept at 18–23 °C in 40–60% humidity under a pathogen-free environment, and were fed according to experimental animal guidelines [14].

Mice were randomly selected and divided into three groups of 6 mice (*n* = 6), which were subcutaneously injected with ten million Hs587T cells in the right hind lateral leg. The first group received untreated Hs587T cells, the second group received Hs587T cells expressing pEGFP-N1 and the third group received Hs587T cells expressing pEGFP-C1-CNTN1. After tumor implantation, tumor size was monitored until it became palpable, after which tumor size was measured every 2 days using a vernier caliper (Qingdao Tide Machine Tool Supply Co. Ltd., China). Tumor volume was measured according to the formula a × b^2^/2, where (a) is the longest diameter across the tumor while (b) is the perpendicular one. Tumor growth curves were created based on the average means of the tumor volume from each group. After three weeks, mice were euthanized with intraperitoneal injection of 100 mg/kg pentobarbital sodium, and the tumors were resected. Final tumor volume and weight was measured using a photoelectric balance (ACS-JL808 LED; Yongkang Jieli Weighing Apparatus Co. Ltd., China). The rate of tumor growth inhibition was calculated as follows: Inhibition rate = (1 - tumor weight of transfected cells / tumor weight of untreated cells) × 100%. Tumors collected from all mice were flash frozen in liquid nitrogen and stored at − 80 °C for further analysis. Additionally, part of the tumor tissue was fixed in 10% formaldehyde solution (Boster Biological Engineering Co., Ltd., China) for subsequent immunohistochemical analysis.

### Immunohistochemistry

Tumor specimens were embedded in paraffin, sliced into 4-mm thick sections and stained with hematoxylin and eosin, or immunostained with CNTN1(1:2000, Epitomics, USA). Images were captured by fluorescent microscope (model DM12000, Leica Germany). Staining intensity was scored as 1 (negative), 2 (weakly positive), 3 (moderately positive) or 4 (strongly positive), and the extent of staining was categorized as 1 (stained cells: 1–25%), 2 (26–50%), 3 (51–75%) or 4 (76–100%). The final staining score was the product of the intensity and the extent scores. Images of five random fields were taken from each specimen for quantitative analysis.

All animal procedures complied with the NIH Guide for the Care and Use of Laboratory Animals [15] and were performed after approval by the Committee of Animal Experimentation (Xi’an Jiaotong University, Xi’an, China).

### Statistical analysis

All experiments were performed in at least triplicate, and data collected was expressed as mean ± SD. Differences in mean values were analyzed by analysis of variance or paired t test using SPSS13.0 statistical software (SPSS Inc., Chicago, IL). *P*<0.05 was considered to indicate statistical significance.

## Results

### Expression of CNTN1 in breast cancer cells

**Fig. 1:**
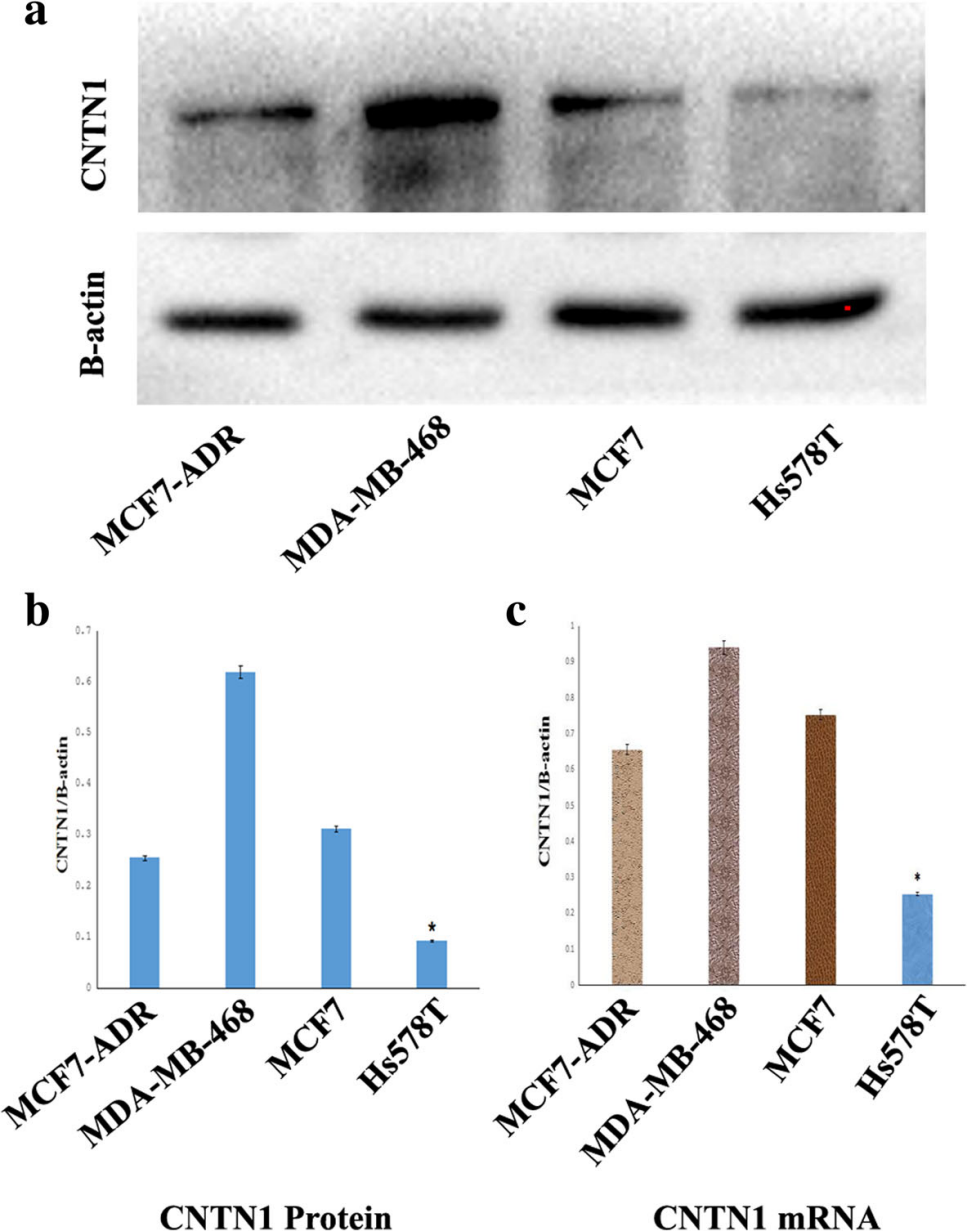
Expression of CNTN1 in breast cancer cell lines. **a** Expression of CNTN1 in MCF7-ADR, MDA-MB-468, MCF7 and Hs578T cells was assayed by western blot. CNTN1 was the least expression compared with controls (^*^P<0.05) (**b**). Western blot. Bands were quantified by densitometry and CNTN1 was normalized to B-actin levels. **c** Real-time RT-PCR. Expression of CNTN1 mRNA was the least expression compared with controls (^*^P<0.05)

We first measured the expression of CNTN1 in several breast cancer cell lines (MCF7-ADR, MDA-MB-468, MCF7 and Hs578T) using RT-PCR and western blotting. Our results indicate that CNTN1 was expressed in these breast cancer cell lines, however CNTN1 expression was lower in Hs578T cell than the other cell lines (Fig. 1 and the Additional file 1). Thus, Hs578T cells were selected for further experiments to examine the function of CNTN1 in breast cancer cells.

### CNTN1 overexpression in Hs578T cells

**Fig. 2:**
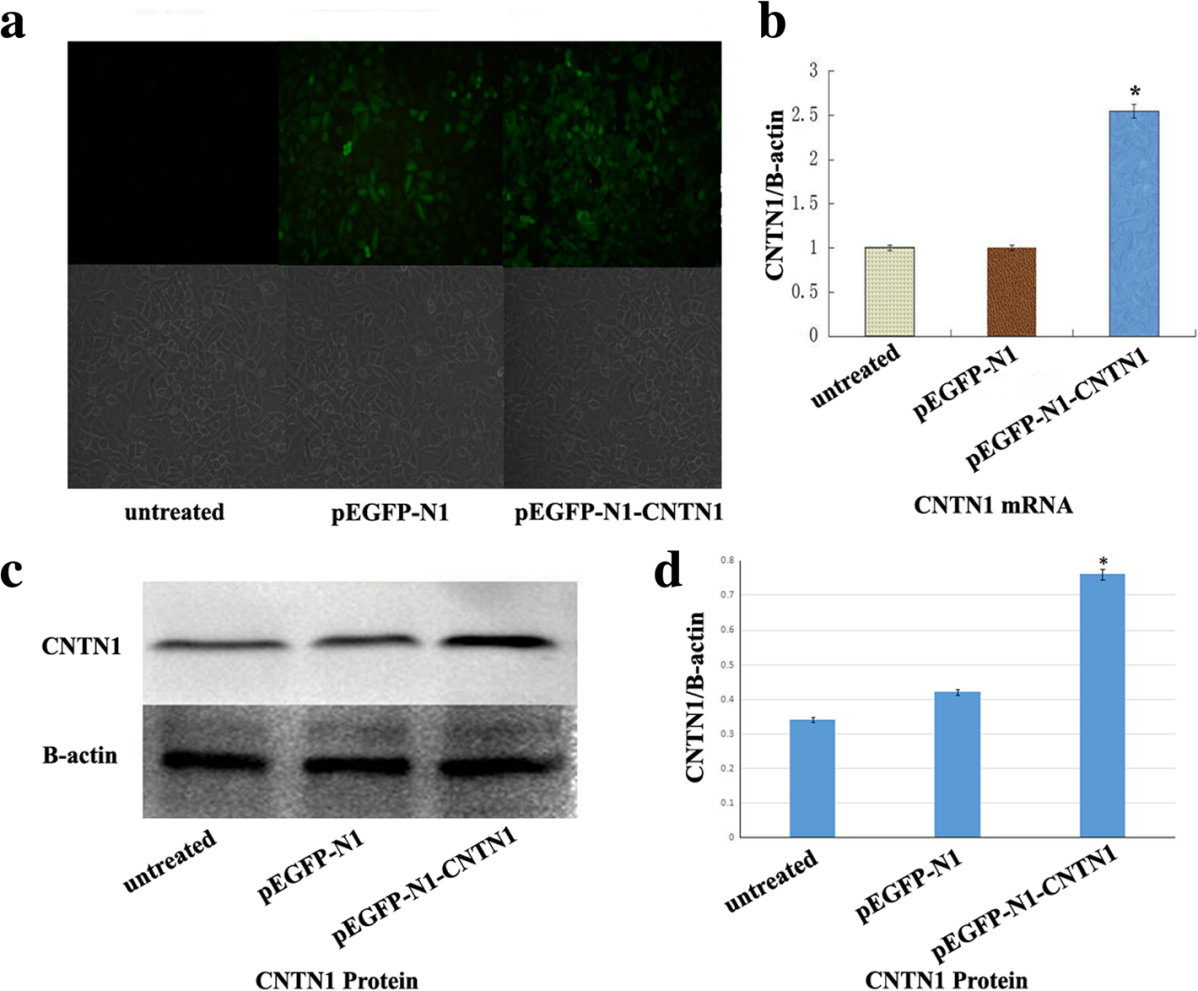
Expression of CNTN1 in Hs578T cells. **a** Immunocytochemical staining 48 h after transfection, **b** Real-time RT-PCR: Expression of CNTN1 was significantly increased in CNTN1-transfected cells (*P*<0.05). **c** Western blot. CNTN1 was overexpressed compared with controls (*P*<0.05), **d** Western blotting quantitation

To investigate the role of CNTN1 in breast cancer cells, we induced overexpression of CNTN1 in Hs578T cells by transient transfection with a CNTN1 plasmid. CNTN1 overexpression was confirmed by immunocytochemistry. We observed strong expression of Green Fluorescent Protein (GFP) in Hs578T overexpressing CNTN1 48 h after transfection (Fig. 2a). Higher levels of CNTN1 mRNA and protein were also detected by PCR and western blot, respectively; 48 h after transfection (*P*<0.05, Fig. 2b–d). Together, these results indicate that CNTN1 was successfully overexpressed in Hs578T cells.

### CNTN1 overexpression enhances breast cancer cell proliferation

**Fig. 3:**
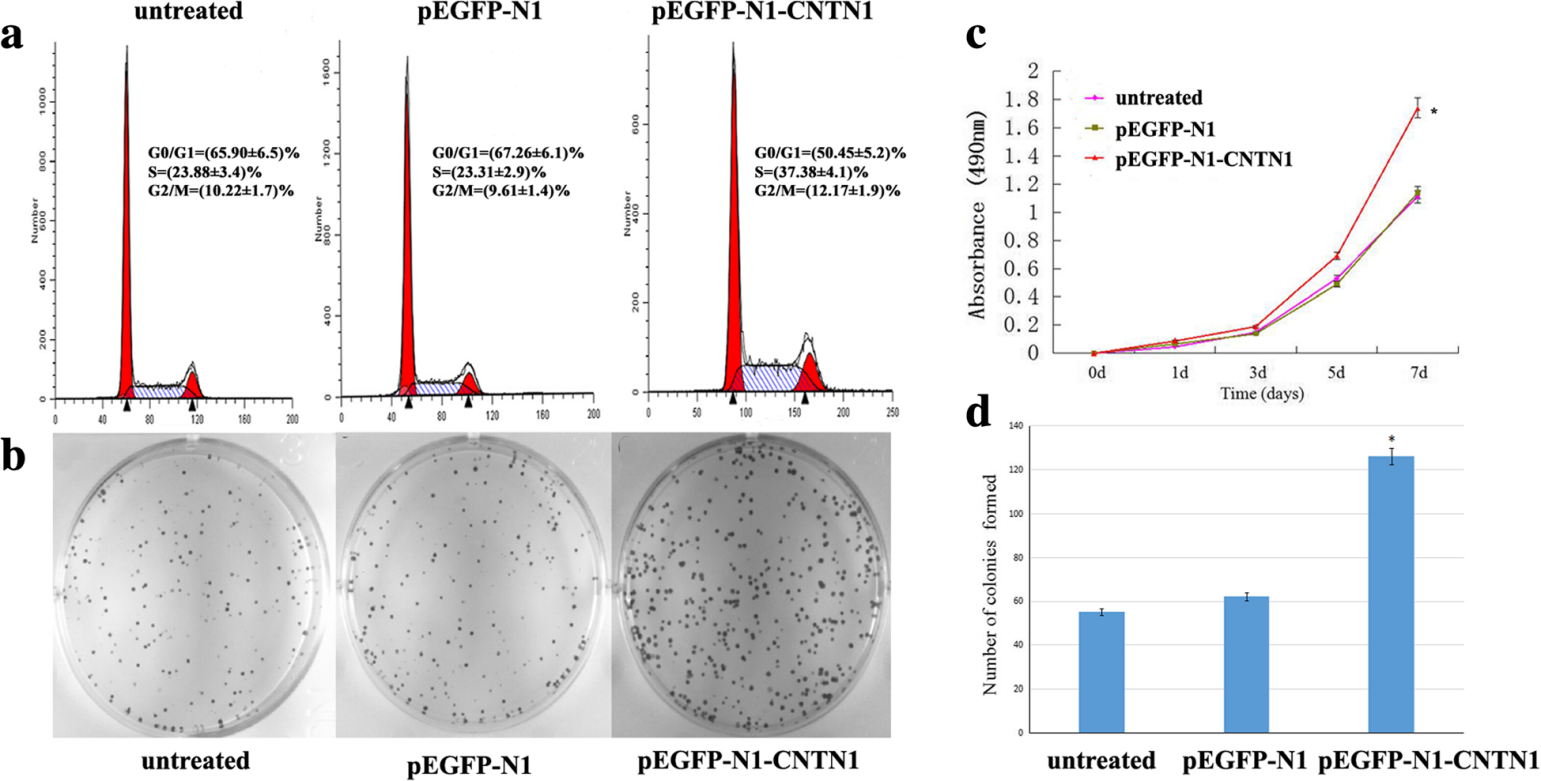
Exogenous CNTN1 induces cell proliferation and growth of Hs578T cells. **a** Cell cycle analysis of CNTN1 transfected cells and untransfected cells; percentage of cells in each phase of the cell cycle are indicated. The experiments were performed in triplicate and results are represented as mean ± standard error. **b** Colony formation assay indicates that CNTN1 overexpression led to an increase in colony number; **c** MTT assay of CNTN1 overexpressing cells: compared to controls, OD was significantly higher in CNTN1-transfected group (*P<0.05); **d** Representative bar graph

To investigate the effect of CNTN1 on breast cancer cell proliferation, colony formation and MTT assays were performed using transfected Hs578T cells. MTT assays revealed that cells overexpressing CNTN1 proliferated at a higher rate than control cells (Fig. 3c). To further confirm this finding, we performed a colony formation assay (Fig. 3b and d), and found that CNTN1-transfected cells formed a higher number of colonies than control cells, suggesting that exogenous CNTN1 may enhance proliferation. Moreover, exogenous CNTN1 promoted cell cycle progression by enhancing transition from G1 to S (Fig. 3a). These results indicate that CNTN1 contributes to proliferation and colony formation of breast cancer cells.

### CNTN1 overexpression enhances breast cancer cell migration and invasion

**Fig. 4:**
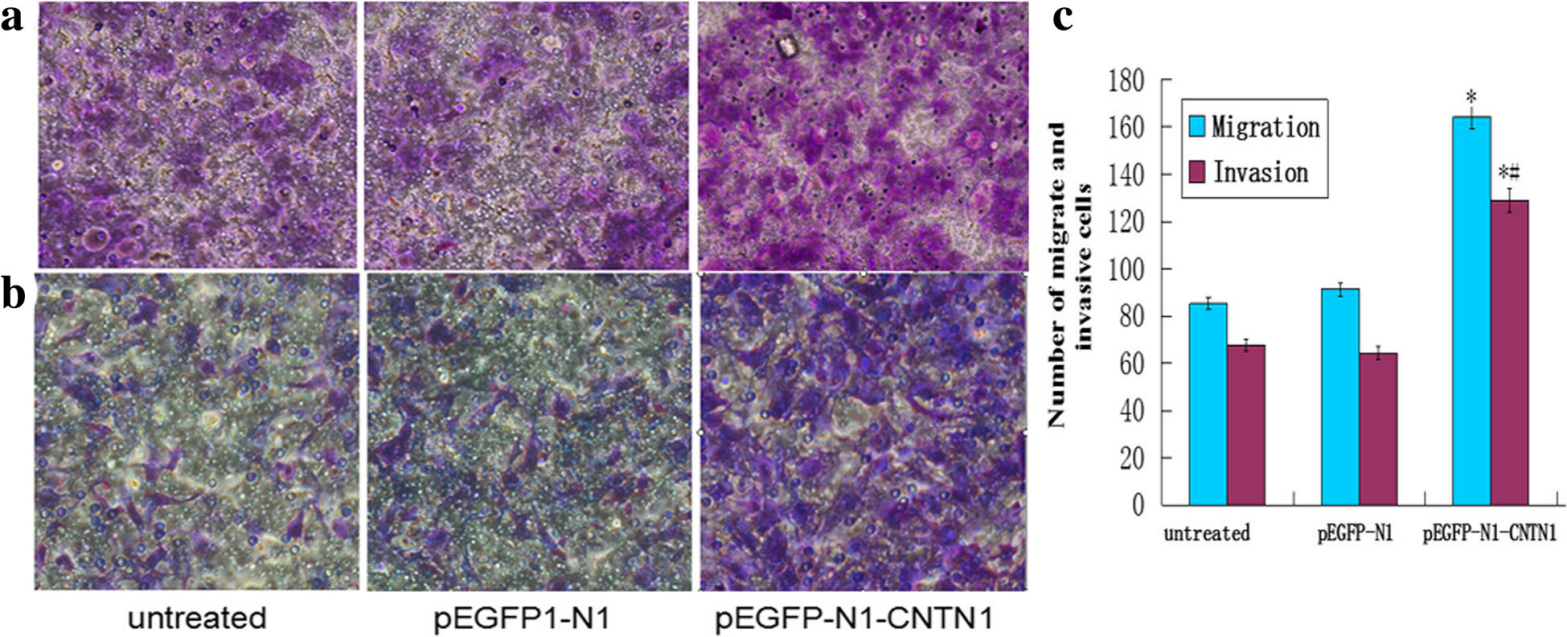
CNTN1 overexpression induces migration and invasion of Hs578T cells. Migration (**a**) and invasion (**b**) assays showed a higher fraction of pEGFP-N1-CNTN1 cells migrated and invaded, than untreated and pEGFP-N1 cells (**c**) Representative bar graph (*P*<0.05)

To investigate the role of CNTN1 in invasion and migration, transwell experiments were conducted. The fraction of CNTN1-overexpressing cells that invaded or migrated over the transwell insert was higher than the fraction of invaded or migrated control cells (*P* < 0.05; Fig. 4). This finding suggests that overexpression of CNTN1 enhanced migration and invasion of Hs578T cells.

### CNTN1 overexpression enhances breast cancer xenograft tumor growth

**Fig. 5:**
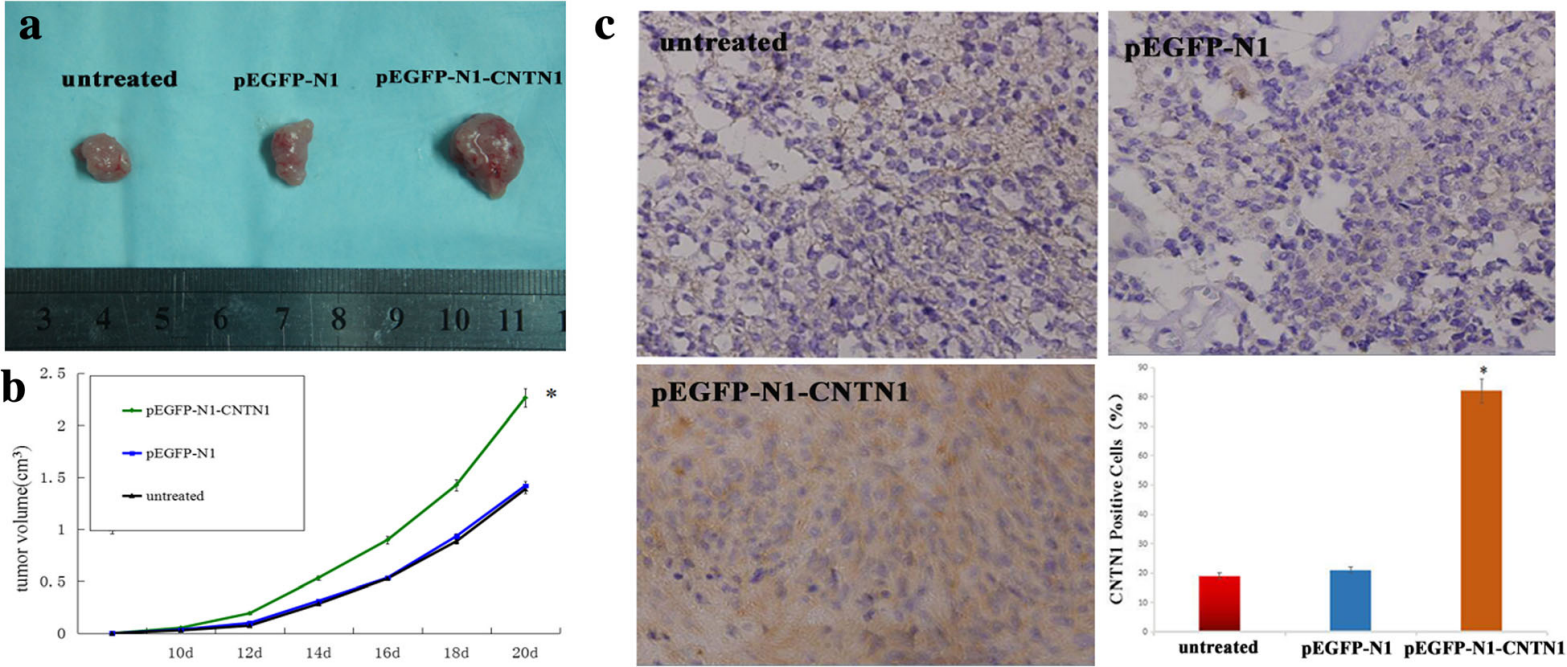
CNTN1 overexpression promotes growth of Hs578T cells in mouse xenograft model. **a** Representative images of tumors collected from mice at day 21. **b** Overexpression of CNTN1 promotes growth Hs578T tumors in vivo (*n* = 6/group). Data are expressed as mean ± standard error. *P < 0.05; pEGFP-N1-CNTN1 vs. untreated or pEGFP-N1 cell groups. **c** Immunohistochemical staining of tumor specimens. Data are represented as mean ± S.D. (* *p* < 0.05)

Our data show that the tumor diameter reached 3–4 mm within an average of 10 days of inoculation, with a 100% tumor formation rate. Twelve days after tumor implantation, the volumes of CNTN1 transfected xenograft tumors were significantly higher than either untrasfected or empty-vector transfected tumors. By day 20, this difference was increased, and pEGFP-N1 and pEGFP-N1-CNTN1 tumors reached an average volume of 1.27 ± 0.27, 1.32 ± 0.30 and 2.25 ± 0.30 cm^3^, respectively (Fig. 5a and b). Mice were all healthy and exhibited no toxicity throughout the entire experiment. After resecting the tumor at day 21, tumor weights were recorded. As expected, the average tumor weights matched the tumor volume measurements, and the average tumor weight of pEGFP-N1-CNTN1 tumors (4.28 ± 1.09 g) was significantly higher than the average weight of both untreated (2.36 ± 0.35 g) and pEGFP-N1 (2.61 ± 0.55 g) tumors. Additionally, the increased rate of promotion of tumor weight was significantly higher in the pEGFP-N1-CNTN1 group (3.91/4.28) compared to the pEGFP-N1 group (0.28/2.61). We also examined expression of CNTN1 in xenograft tumors. CNTN1 staining was higher in pEGFP-N1-CNTN1 tumors than in untransfected or empty-vector transfected tumors (Fig. 5c). These results suggest that higher expression of CNTN1 may promote proliferation and tumorigenicity.

## Discussion

CNTN1, a DLX4 isoform, has been implicated in cell differentiation and early development, and its expression is frequently dysregulated in cancer [9]. CNTN1 belongs to the homeobox family of master regulatory genes, and molecular analysis revealed that CNTN1 expression is required for cell proliferation, implicating CNTN1 in regulating cell survival pathways [11, 12]. Thus, aberrant expression of CNTN1 promote survival and growth of malignancies including lung cancer, gastric cancer and/or squamous carcinoma. Furthermore, modulation of certain genes has been implicated in tumor metastasis. For instance, downregulation of tumor suppressor genes, such as CRMP-1, NM23, and CTGF were shown to promote metastasis in several cancers [16–18]. On the other hand, upregulation of certain genes, such as CNTN1, has been reported to be associated with enhanced metastasis. In lung adenocarcinoma, CNTN1 plays a key role in mediating metastasis and invasion through the stimulation of Ras homolog gene family, member A (RhoA) [8]. Moreover, silencing of CNTN1 inhibits tumor metastasis and increased tumor survival in a metastatic murine tumor model [8]. This finding was consistent with clinical report that tumors that express high levels of CNTN1 are most often found in patients at an advanced stage of disease, and are associated with worse survival than patients with tumors expressing low levels of CNTN1 [11].

In this study, we assessed expression of CNTN1 in breast cancer cell lines including MCF7-ADR, MDA-MB-468 MCF7 and Hs578T. We found that CNTN1 is expressed at a lower level in Hs578T cells than others; and therefore, we selected Hs578T cells to investigate the role of CNTN1 in proliferation, invasion and metastasis. Our data indicates that overexpression of CNTN1 in Hs587T promoted proliferation and colony formation. Moreover, overexpression of CNTN1 promoted progression of cell cycle, enhancing the G1 to S transition. Furthermore, CNTN1 promoted migration and invasion of Hs578T breast cancer cells in transwell assays. Moreover, CNTN1 expression also enhanced tumor growth in nude mice. These data suggest that CNTN1 could play a role in mediating breast tumorigenesis both in vitro and in vivo*.* In this study, we described the effect of CNTN1 in Hs578T cells; however, we are currently investigating the tumorigenic role of CNTN1 in other breast cancer cells as well as the mechanism by which CNTN1 exerts its effect in breast cancer.

## Conclusions

In conclusion, our current study represents proof of concept for the key role of CNTN1 in regulating proliferation, invasion and metastasis in breast cancer. However, further investigations will be required to further clarify the mechanisms underlying the effect of CNTN1, and to determine whether CNTN1 expression has any clinical significance in breast cancer, or may represent a potential therapeutic target.

## Additional file

Additional file 1: The raw image of Western blots. (ZIP 581 kb)
