# Supplementary figures and images for: Overexpression of Contactin 1 promotes growth, migration and invasion in Hs578T breast cancer cells
Source: BMC Cell Biol. 2018 Apr 19;19:5. doi: 10.1186/s12860-018-0154-3 (PMC5907708; doi:10.1186/s12860-018-0154-3)

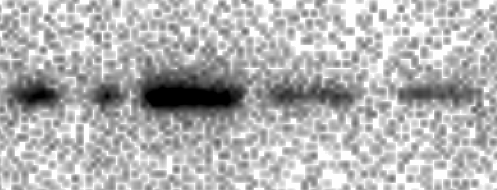

Supplement: Supplementary file 1 — The raw image of Western blots. (ZIP 581 kb) [file 12860_2018_154_MOESM1_ESM.zip › 6.tif]

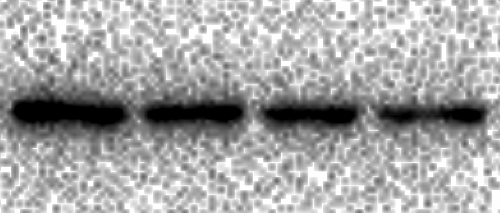

Supplement: Supplementary file 1 — The raw image of Western blots. (ZIP 581 kb) [file 12860_2018_154_MOESM1_ESM.zip › 6a.tif]

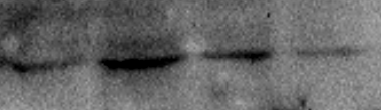

Supplement: Supplementary file 1 — The raw image of Western blots. (ZIP 581 kb) [file 12860_2018_154_MOESM1_ESM.zip › 7.tif]

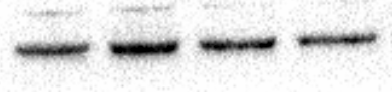

Supplement: Supplementary file 1 — The raw image of Western blots. (ZIP 581 kb) [file 12860_2018_154_MOESM1_ESM.zip › 7a.tif]

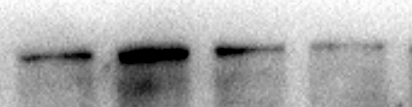

Supplement: Supplementary file 1 — The raw image of Western blots. (ZIP 581 kb) [file 12860_2018_154_MOESM1_ESM.zip › 8.tif]

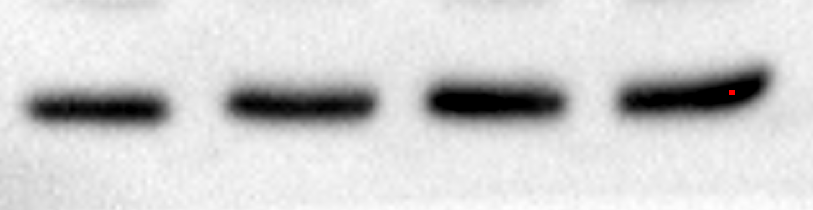

Supplement: Supplementary file 1 — The raw image of Western blots. (ZIP 581 kb) [file 12860_2018_154_MOESM1_ESM.zip › 8a.tif]
